# Is Medicare Home Health Care Utilization Substituting for Long‐Term Care? Evidence From Dual Eligible Beneficiaries

**DOI:** 10.1111/1475-6773.70109

**Published:** 2026-04-01

**Authors:** Mingyu Qi, Rachel M. Werner, Megan Huisingh‐Scheetz, R. Tamara Konetzka

**Affiliations:** ^1^ Department of Public Health Sciences University of Chicago Chicago Illinois USA; ^2^ Leonard Davis Institute of Health Economics, University of Pennsylvania Philadelphia Pennsylvania USA; ^3^ Department of Medicine University of Chicago Chicago Illinois USA

**Keywords:** dual eligible, long‐term care, Medicaid home‐ and community‐based services, Medicare home health care

## Abstract

**Objective:**

To examine the plausibly causal effect of Medicaid home‐ and community‐based services (HCBS) use on Medicare community‐initiated home health care (CIHHC) utilization among dual‐eligible older adults and to provide evidence on whether access to home‐based long‐term care (LTC) reduces use of Medicare home health care, with potential implications for whether Medicare home health care is used as a substitute for LTC services when they are not or less accessible.

**Study Setting and Design:**

To address the endogeneity of Medicaid HCBS use, we employ an instrumental variable, the proportion of Medicaid HCBS enrollment in other counties within the same state in the previous quarter, in conjunction with a state‐border design in estimating the effect of Medicaid HCBS use on Medicare CIHHC and its heterogeneity.

**Data Sources and Analytic Sample:**

We use national Medicare and Medicaid claims data along with home health and nursing home assessment data from 2016 to 2019. Our sample consists of 36,955,226 beneficiary‐quarter‐level observations of older adults (65+) dually enrolled in Medicaid and Medicare and residing in contiguous state‐border counties.

**Principal Findings:**

Medicaid HCBS use reduces Medicare CIHHC utilization by approximately 1.02 percentage points (95% CI: −1.73 to −0.32), representing about 44% of the sample mean. This effect is concentrated among beneficiaries enrolled in Medicare‐Medicaid integrated care plans and those living in urban counties. Moreover, the reduction is most pronounced among older adults who live alone and have around‐the‐clock assistance needs.

**Conclusions:**

Our findings suggest a substitution between Medicaid HCBS and Medicare CIHHC among dual‐eligible older adults at the margin of using Medicaid HCBS, a relationship that should be taken into account when evaluating either program. These findings also align with the hypothesis that Medicare CIHHC is being used as a substitute for LTC, and the availability of home‐based LTC may help to alleviate this potentially inefficient use.

## Callout Box

- What is known on this topic?
  ○ The increase in Medicare community‐initiated home health care (CIHHC) from 2010 to 2020 was driven primarily by growth among Medicare Advantage (MA) enrollees who were not eligible for Medicaid.
  ○ A prior study found that Medicaid HCBS use significantly shifts Medicare post‐acute care utilization away from nursing homes toward home health.
  ○ Despite substantial growth in Medicare CIHHC and Medicaid HCBS over recent decades, little is known about how the use of services from one program affects utilization of the other.
- What this study found?
  ○ This study provides the first plausibly causal national estimate of how Medicaid HCBS use affects Medicare CIHHC utilization.
  ○ Medicaid HCBS use is estimated to decrease Medicare CIHHC utilization by approximately 1.02 percentage points, suggesting a potential substitution between the two programs among dual‐eligible older adults.
  ○ Medicare CIHHC is potentially being used as a substitute for long‐term care, and the availability of home‐based long‐term care services may help to alleviate this potentially inefficient use.

## 1 Introduction

Medicare covers home health services for beneficiaries who are homebound and need short‐term skilled nursing care or therapy services. Unlike the skilled nursing facility (SNF) benefit, Medicare does not require a preceding hospital stay for beneficiaries to qualify for home health. In 2019, about 3.3 million Traditional Medicare (TM) beneficiaries received home health care, with approximately 66% of these episodes being community‐initiated home health care (CIHHC), defined as episodes not preceded by an inpatient stay [1]. This proportion was 53% in 2001, representing an increase of about 25% over two decades.

During the same period, home‐based care programs provided through Medicaid‐funded home‐ and community‐based services (HCBS) has also grown substantially. HCBS includes non‐skilled, lower‐cost services like assistance with activities of daily living (ADLs) that can be provided indefinitely to those who qualify. Medicaid is the primary payer for long‐term care (also referred to as long‐term services and support (LTSS)) in the United States. For those who qualify due to low income and assets in conjunction with need, Medicaid covers both institutional care (e.g., nursing homes) and home‐ and community‐based care. As HCBS are generally less costly for state Medicaid programs and better align with older beneficiaries' preference to age at home, Medicaid has been shifting its LTSS toward a higher proportion of HCBS over the past few decades. By 2020, around 72% of Medicaid beneficiaries who used LTSS received services in home‐ and community‐based settings [2]. While all states cover HCBS to some degree, the extent varies considerably across states [3, 4]. In all states, access to HCBS services among older adults (65+) is restricted to a small fraction who are dually enrolled in Medicare and Medicaid (dual‐eligible), representing approximately 15% of older adults in the United States [5].

A recent study examining trends in Medicare home health use from 2010 to 2020 found that the increase in Medicare CIHHC was driven by growth among non‐dual‐eligible Medicare Advantage (MA) enrollees [6]. Although the Medicare home health benefit generally does not cover LTSS, it can provide help with ADLs when the beneficiary is concurrently receiving skilled services, and the skilled services themselves may temporarily address needs associated with long‐term care. The Medicare home health skilled services are short‐term, typically ≤ 21 days in duration, but can be renewed by providers after a 60‐day period if the patient has additional qualifying medical diagnoses [7]. For those who do not qualify for Medicaid, home health care may be the only in‐home intervention available to older adults needing more support. The significant growth in Medicare CIHHC use among non‐dual‐eligible beneficiaries, who lack access to publicly funded LTSS such as Medicaid HCBS, raises concern that Medicare home health may increasingly be used to fill unmet LTSS needs at home. This substitution may be particularly likely among MA enrollees because some MA plans have greater flexibility in covering nonskilled services through the home health benefit [8]. While we cannot directly examine substitution between Medicare CIHHC and long‐term care needs, we seek to inform this hypothesis by assessing whether Medicaid HCBS utilization helps offset the utilization of Medicare CIHHC among dual‐eligible beneficiaries.

Despite substantial growth in both Medicare CIHHC and Medicaid HCBS over recent decades, rigorous evidence on how the use of services from one program affects the utilization of the other remains scant (Appendix Table 1). One study investigated the interaction between Medicaid HCBS and Medicare post‐acute care (PAC) use, finding that dual‐eligible beneficiaries using Medicaid HCBS had higher use of home health care relative to nursing home care, conditional on receiving PAC [9]. There have also been evaluations differentiating characteristics of individuals receiving home health care referrals from community providers versus referrals from hospitals or SNFs. Medicare CIHHC users are older, more likely to be dual eligible, have multiple home health episodes, have Alzheimer's disease, and have greater needs for informal caregiving than those referred from hospitals or SNFs [10, 11, 12, 13]. To date, the only study that has explicitly explored potential substitution between Medicare CIHHC and LTSS is a 2014 report, which did not find evidence of such an effect [14]. However, that study was descriptive and did not account for potential selection bias in LTSS use. If people who are sicker in unmeasured ways use more of both services, any estimate of substitution between the two services will be biased toward zero.

Our study fills this gap in the evidence by assessing how Medicaid HCBS use affects Medicare CIHHC utilization among dual‐eligible older adults using national data. To obtain plausibly causal estimates, we employ a novel instrumental variable that exploits variation in state‐level Medicaid HCBS enrollment rates, calculated without including the index county, in combination with a state‐border‐county design. We hypothesize that Medicaid HCBS use decreases Medicare CIHHC utilization among dual‐eligible older adults, potentially implying that addressing LTSS needs directly will decrease the need to use Medicare CIHHC. By assessing the relationship between Medicare CIHHC and Medicaid HCBS, our results help inform whether Medicare home health care may be used as a substitute for long‐term care, with implications for future policy reforms in both Medicare and Medicaid that target home‐based services.

## 2 Methods

### 2.1 Data

Our primary data source is the Master Beneficiary Summary File (MBSF), which contains individual‐level Medicare and Medicaid enrollment and demographic information for all Medicare beneficiaries. We supplement the MBSF data with the Outcome and Assessment Information Set (OASIS) data, the Medicare Provider Analysis and Review (MedPAR) data, and the Minimum Dataset (MDS) to measure Medicare CIHHC use. OASIS and MDS data contain federally required assessment information for patients receiving services from Medicare‐ or Medicaid‐certified home health agencies and nursing homes, respectively. MedPAR contains discharge‐level inpatient Medicare claims. Specifically, we use OASIS to capture all home health episodes during the study period. Both MedPAR and MDS are used to determine whether a home health episode was initiated from the community. We then merge our data set with Transformed Medicaid Statistical Information System Analytic Files (TAF), which include enrollment, service utilization, and claims for Medicaid enrollees, to identify Medicaid HCBS use. Specifically, we pull waiver enrollment information from the TAF Demographic and Eligibility files and information on the use and type of HCBS from the TAF Other Services files. In Appendix Table 2, we report additional details following the TAF Reporting Checklist [15]. Finally, we incorporate home health agency (HHA)‐level characteristics from the Provider of Services files and zip code‐level socioeconomic covariates derived from the 2015–2019 American Community Survey 5‐Year Estimates.

### 2.2 Sample

We include all Medicare beneficiaries aged 65 and older between 2016 and 2019, during which time there were no significant changes to Medicare home health benefit eligibility. To create time windows for identifying Medicaid HCBS and Medicare CIHHC use, we construct the sample at the individual‐quarter level. We exclude data from the fourth quarter of 2019 because the number of OASIS records in December 2019 is significantly lower than in prior years, indicating a potential data quality issue. As Medicaid HCBS are only available to dual‐eligible beneficiaries, we restrict the sample to those who were fully dual‐eligible at any point during the study period. By including quarters prior to a beneficiary becoming fully dual‐eligible, the sample better represents beneficiaries near the margin of Medicaid eligibility. This approach helps mitigate potential selection bias if Medicaid enrollment was prompted by care needs that also increase the likelihood of HCBS use.

**FIGURE 1:**
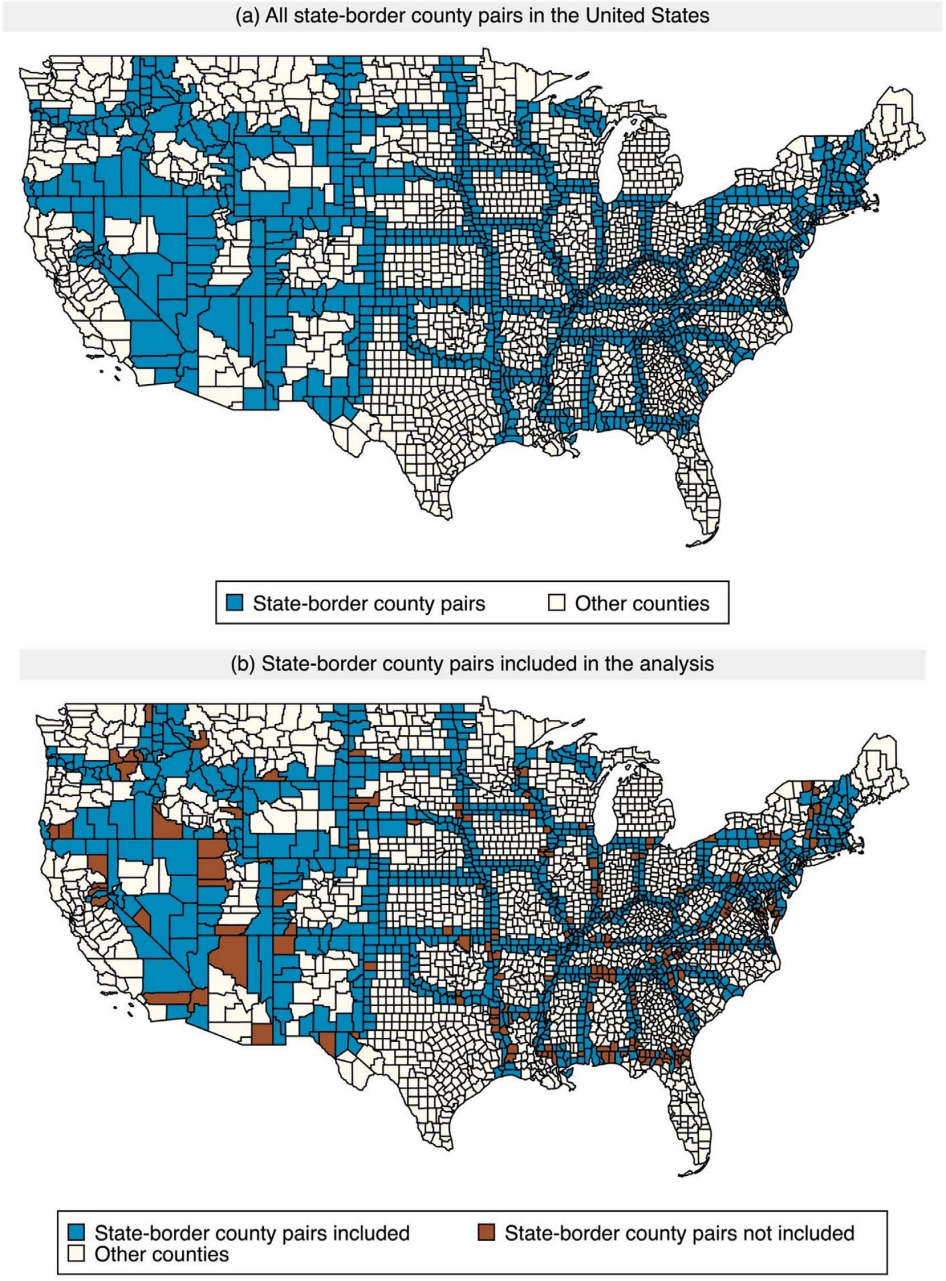
All state‐border county pairs in the United States and those included in the analysis.

Our empirical strategy exploits variation in state‐level Medicaid HCBS enrollment. To improve comparability across beneficiaries who are subject to different state policies on HCBS coverage, we limit the sample to individuals residing in state‐border counties as described in Section 2.4 [16, 17]. Of the 3108 counties in the contiguous United States, 1184 lie along state borders. Because some counties have multiple out‐of‐state neighbors, a county may appear in the sample multiple times, yielding 1272 unique pairs after excluding those separated by large bodies of water (see Figure 1a). We further restrict our sample to beneficiaries living in state‐border county pairs with the same urban–rural classification to improve the balance of sociodemographic characteristics and service supply within each pair. As our empirical strategy relies on variation in state‐level Medicaid HCBS enrollment, we exclude beneficiaries living in Washington, D.C. Finally, we exclude observations of beneficiaries who switched between TM and MA, moved to a different county, lacked eligible days for CIHHC in any quarter, or had missing values for any analysis variables. Our final sample includes 36,955,226 beneficiary‐quarter observations from 884 unique state‐border county pairs (see Figure 1b). A flow diagram illustrating the construction of the study sample is presented in Appendix Figure 1.

### 2.3 Study Variables

#### 2.3.1 Outcome Variable

The outcome variable is whether the beneficiary used any Medicare CIHHC during a given quarter. Following MedPAC's definition, we define a home health episode as community‐initiated if the beneficiary had no inpatient claim or MDS assessment in the 14 days before the start of the episode [1].

#### 2.3.2 Treatment Variable

The treatment variable is whether a beneficiary was enrolled in a 1915(c) aged or aged/disabled HCBS waiver and/or received any Medicaid HCBS services (excluding transportation‐only), either through a 1915(c) waiver or through state plan offerings, during the previous quarter [9, 18]. We refer to beneficiaries meeting this criterion as prevalent HCBS users. Specific data sources and elements used to identify HCBS are presented in Appendix Table 4.

#### 2.3.3 Covariates

We control for individual‐level characteristics including age, sex, race and ethnicity, original reason for Medicare entitlement (old age vs. disability/end‐stage renal disease (ESRD)), Medicare type (TM vs. MA), and number of days eligible for Medicare CIHHC per quarter to adjust for potential disparities in care access and outcomes. We also control for zip code‐level socioeconomic characteristics, such as median household income, percentage of population over 65 living in poverty, and other related factors. To account for local supply of home health services, we include the number of HHAs, registered nurses, and home health aides per 1000 beneficiaries at the county level. Finally, we control for the county‐year MA penetration rate, calculated using the MBSF.

### 2.4 Empirical Strategy

We aim to estimate the causal effect of Medicaid HCBS use on Medicare CIHHC utilization. However, Medicaid HCBS users may differ from non‐users in unobserved ways that also affect Medicare CIHHC use. For instance, individuals with more complex conditions may be more likely to use both Medicaid HCBS and Medicare CIHHC, potentially biasing the estimated relationship between the two programs. To address potential endogeneity, we employ a “leave‐out” mean instrumental variable (IV) approach, commonly used in health economics and health services research [19, 20, 21, 22, 23], in conjunction with our state‐border‐county strategy.

Our instrument is the proportion of prevalent Medicaid HCBS users among dual‐eligible older adults in other counties within the same state in the previous quarter. This proportion reflects state Medicaid funding policy that influences the availability of HCBS. Therefore, it is likely a good predictor of individual‐level HCBS use (*first‐stage effect*). We assess the correlation between the IV and HCBS use by examining whether the *F*‐statistics exceed the standard threshold [24, 25]. Because our IV is measured among dual‐eligible older adults living in *other* counties within the same state in the previous quarter, it is unlikely to be correlated with individual‐ or county‐level characteristics in the focal county that may also affect Medicare CIHHC use in the current quarter (*exclusion restriction*). We acknowledge that our instrument may still be vulnerable to unobserved state or regional shocks that can influence both HCBS and CIHHC use. We therefore strengthen our IV strategy by applying it to state‐border county pairs. These counties constitute reasonable comparison groups, as a county is likely to share similar socioeconomic conditions, home health providers, and market dynamics with its geographically proximate cross‐border counterpart, thereby mitigating the impact of unobserved state or regional shocks.

The exclusion restriction assumption is fundamentally untestable; however, we conduct an indirect test by comparing population characteristics at the county‐year level within each pair. The instrument is less likely to be correlated with unobserved confounders if these characteristics are similar between the paired counties. Finally, a valid instrument should affect all individuals in the same way (*monotonicity*). We assess this assumption by summarizing the mean Medicaid HCBS use across quartiles of the instrument.

We first estimate a multivariable regression using ordinary least squares (OLS). Specifically, we estimate the following equation:

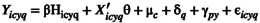




The subscripts i, c, y, q denote individual, county, year, and quarter, respectively. $Y_{\text{icyq}}$ represents Medicare CIHHC use. 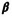 captures the average treatment effect of prevalent Medicaid HCBS use on Medicare CIHHC utilization when endogeneity is addressed. $X_{\text{icyq}}^{\prime }$ represents the individual‐level covariates. We include county fixed effects $\mu_{c}$ to control for time‐invariant county‐specific confounders, quarter fixed effects $\delta_{q}$ to account for seasonal trends, and state‐border county‐pair‐year fixed effects $\gamma_{\mathrm{py}}$ to address time‐varying confounders that affect paired counties similarly. Standard errors are clustered separately at the county and state‐border county‐pair levels.

Next, we implement the IV regressions using two‐stage least squares (2SLS), controlling for the same set of covariates and fixed effects in both stages. With the inclusion of county and state‐border county‐pair‐year fixed effects, the model is identified through variation in the instrument both within counties over time and between counties in each state‐border county pair each year.

To assess potential effect heterogeneity, we repeat the IV analysis across sample subgroups. We first stratify by Medicare type. Because MA plans have strong incentives to reduce Medicare spending, they are likely to limit Medicare CIHHC use when Medicaid HCBS are available. We therefore hypothesize that Medicaid HCBS use has a larger effect on Medicare CIHHC utilization among MA enrollees. Second, since Medicare‐Medicaid integrated care plans (ICPs) may offer better care coordination between the two programs and may put MA plans at risk for both Medicaid and Medicare spending [26], we stratify by enrollment in such plans, including Dual Eligible Special Needs Plans and Financial Alignment Initiatives, among MA enrollees. Third, we stratify by original reason for Medicare entitlement, reflecting differences in home‐based care needs between individuals aged into Medicare and those eligible due to disabilities or ESRD. Fourth, we stratify by urban versus rural counties to account for potential disparities in access to home‐based care between urban and rural areas.

Lastly, to examine heterogeneity by living arrangements and availability of non‐agency caregivers (family members, friends, or private‐paid help), we classify Medicare CIHHC users into five mutually exclusive categories based on item M1100 (Patient Living Situation) in OASIS and examine the effect of Medicaid HCBS use on each category. Specifically, we classify users into the following groups: living alone in a private home with around‐the‐clock assistance, living alone in a private home without around‐the‐clock assistance, living with others in a private home with around‐the‐clock assistance, living with others in a private home without around‐the‐clock assistance, and living in a congregate setting (e.g., assisted living facility). If Medicare CIHHC is, to some extent, used as a substitute for long‐term care, we hypothesize that Medicaid HCBS is more likely to decrease home health use for older adults with greater LTSS needs—specifically, those who live alone and require around‐the‐clock assistance.

We conduct three additional analyses to assess the robustness of our study design. The instrument we use reflects state‐level Medicaid LTSS policies and infrastructure, raising the concern that it may correlate with other state‐level confounders that also affect Medicare CIHHC use. Therefore, we perform a falsification test by regressing Medicare CIHHC use on the instrument among non‐dual‐eligible older adults, who are not eligible for Medicaid HCBS. A null effect provides further evidence that our instrument affects the use of Medicare CIHHC only through Medicaid HCBS use. To examine the sensitivity of our results to the choice of the state‐border county‐pairs sample, we repeat our main analysis in a separate sample that includes all US counties, controlling for the same set of covariates, and county, year, and quarter fixed effects. While our preferred sample prioritizes internal over external validity, this sample does the opposite. Finally, as prior studies suggest that Medicaid HCBS claims in some states have substantial data quality limitations during our study period, we repeat our main analysis in a subsample that excludes states (Alabama, Hawaii, Iowa, Nebraska, Nevada, South Carolina, Wisconsin, North Dakota, Oregon, and Washington) in which the rate of HCBS identification among 1915(c) waiver claims is below 90% [18].

We conduct all analyses using SAS version 9.4 and Stata version 16.0.

## 3 Results

### 3.1 Descriptive Analysis

**FIGURE 2:**
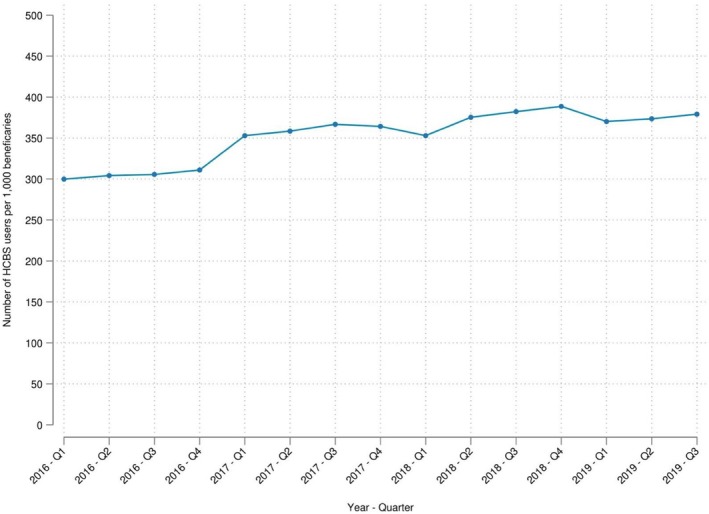
Trend in the utilization of Medicaid HCBS in the state‐border county‐pairs sample. HCBS, home‐ and community‐based services.

Figure 2 depicts the trend in Medicaid HCBS use, showing a steady increase over the study period. A similar trend is observed in the all‐counties sample (see Appendix Figure 2).

We present descriptive statistics of the study sample by Medicaid HCBS user status in Table 1. Our sample includes 36,955,226 beneficiary‐quarter observations, of which 26,234,565 (about 71%) are Medicaid HCBS users. On average, Medicaid HCBS users are more likely to be female, less likely to be White, less likely to first qualify for Medicare based on age, less likely to enroll in MA, more likely to live in zip codes with lower median household incomes, and more likely to live in rural counties. As a robustness check, we repeat this analysis in the all‐counties sample and find similar results (Appendix Table 5).

**TABLE 1:** Characteristics of the study sample by Medicaid HCBS user status.

	All	Non‐HCBS users	HCBS users
	*N* = 36,955,226	*N* = 26,234,565	*N* = 10,720,661
Age, mean (SD)	76.98 (8.63)	76.65 (8.69)	77.80 (8.45)
Female, %	66.59	65.04	70.40
Race and ethnicity, %			
White	58.56	60.14	54.69
Black or African American	21.07	19.96	23.80
Hispanic	8.29	8.34	8.18
Other	12.08	11.56	13.33
Aged into Medicare, %	75.08	77.84	68.33
Enrolled in Medicare Advantage, %	39.21	39.86	37.61
Eligible days for Medicare CIHHC use, mean (SD)	88.73 (8.70)	88.99 (8.27)	88.09 (9.63)
Median household income of the zip code, mean (SD)	61,482.29 (28,064.64)	62,579.19 (28,367.42)	58,798.05 (27,123.10)
Percent of zip code population of age 65 and over, mean (SD)	15.96 (5.21)	16.02 (5.25)	15.82 (5.12)
Percent of zip code population of age 65 and over that are living in poverty, mean (SD)	13.28 (8.73)	12.78 (8.53)	14.49 (9.08)
Percent of zip code population of age 65 and over with private health insurance, mean (SD)	52.37 (14.83)	53.12 (14.75)	50.52 (14.85)
Number of HHAs in the county per 1000 Medicare beneficiaries, mean (SD)	2.45 (1.73)	2.44 (1.71)	2.48 (1.77)
Number of RNs in the county per 1000 Medicare beneficiaries, mean (SD)	4.07 (4.63)	4.04 (4.72)	4.16 (4.42)
Number of home health aides in the county per 1000 Medicare beneficiaries, mean (SD)	2.33 (3.30)	2.30 (3.15)	2.40 (3.64)
Rural county, %	15.83	15.18	17.43

*Note:* Study sample: state‐border county‐pair sample. The characteristics are summarized at the beneficiary‐quarter level.

Abbreviations: CIHHC, community‐initiated home health care; HCBS, home‐ and community‐based services; HHA, home health agency; RN, registered nurse.

An unadjusted comparison shows a large difference in Medicare CIHHC use between Medicaid HCBS users and non‐users. Specifically, an average of 4.15% of HCBS users receive Medicare CIHHC, compared to only 1.54% of non‐HCBS users (see Appendix Table 6).

### 3.2 Main Analysis

In Table 2, we present estimates of the effects of Medicaid HCBS use on Medicare CIHHC utilization from both multivariable and IV regressions. The multivariable regression results suggest that Medicaid HCBS use increases Medicare CIHHC utilization by approximately 2.54 percentage points, although this estimate is likely biased due to the potential endogeneity discussed earlier. In contrast, the IV estimates indicate that Medicaid HCBS use decreases Medicare CIHHC utilization by approximately 1.02 percentage points, representing a decrease of about 44% from the sample mean. As a validity check, we estimate a reduced‐form regression that directly relates the instrument to Medicare CIHHC utilization and find the result is consistent with the 2SLS estimate. The *F*‐statistic for the first‐stage regression is 173.6, indicating a strong correlation between the instrument and Medicaid HCBS use. Appendix Table 7 presents average Medicaid HCBS use by instrument quartile, showing a monotonic increase with higher instrument values. Appendix Table 8 summarizes observed county‐year characteristics between the two counties in each state‐border pair, indicating that characteristics are well balanced by the instrument.

**TABLE 2:** Effects of Medicaid HCBS use on the utilization of Medicare CIHHC, estimated using ordinary least squares and two‐stage least squares.

	OLS	Reduced form	2SLS
Estimated effect	0.0254*** (0.0049)	−0.0066*** (0.0022)	−0.0102*** (0.0035)
Sample mean	0.023	0.023	0.023
County fixed effects	Yes	Yes	Yes
County‐pair‐year fixed effects	Yes	Yes	Yes
Quarter fixed effects	Yes	Yes	Yes
*F*‐statistic on instrument	—	—	173.6
Estimated first stage effect	—	—	0.65
No. of observations	36,955,226	36,955,226	36,955,226

*Note:* (1) HCBS: home‐ and community‐based services. (2) CIHHC: community‐initiated home health care. (3) OLS: ordinary least squares. (4) Reduced form: regression of Medicare CIHHC utilization directly on the instrumental variable. (5) 2SLS: two‐stage least squares. (6) County‐pair‐year fixed effects: state‐border county‐pair specific year fixed effects. (7) Standard errors clustered at state level and state‐border county‐pair level separately are included in parentheses. (8) *p < 0.10, **p < 0.05, ***p < 0.01.

### 3.3 Heterogeneity

We present results from the stratified analyses in Table 3. When stratified by Medicare type, we find a substantially larger reduction in Medicare CIHHC utilization from Medicaid HCBS use among MA enrollees. In contrast, Medicaid HCBS use does not significantly affect the utilization of Medicare CIHHC among TM enrollees. Within MA enrollees, we further stratify by ICP enrollment, finding that Medicaid HCBS use decreases Medicare CIHHC utilization only among ICP enrollees. Next, we stratify by original reason for Medicare entitlement. We find that Medicaid HCBS use reduces Medicare CIHHC utilization for both groups, with a slightly larger effect among individuals who aged into Medicare. We then stratify by county rural–urban classification and find that Medicaid HCBS use decreases Medicare CIHHC utilization only in urban counties.

**TABLE 3:** Effects of Medicaid HCBS use on the utilization of Medicare CIHHC estimated using two‐stage least squares, stratified by key characteristics.

	Medicare type		Integrated care plans enrollment		Original reason for Medicare entitlement		County rural‐urban classification	
	TM	MA	Non‐enrollees	Enrollees	Disability or ESRD	Old	Rural	Urban
Estimated effect	−0.0061 (0.0048)	−0.0153*** (0.0024)	0.0088 (0.0116)	−0.0119*** (0.0034)	−0.009* (0.0049)	−0.0101** (0.0039)	0.0068 (0.0051)	−0.0156*** (0.0036)
Sample mean	0.0252	0.0195	0.0229	0.017	0.027	0.0216	0.0214	0.0233
County fixed effects	Yes	Yes	Yes	Yes	Yes	Yes	Yes	Yes
County‐pair‐year fixed effects	Yes	Yes	Yes	Yes	Yes	Yes	Yes	Yes
Quarter fixed effects	Yes	Yes	Yes	Yes	Yes	Yes	Yes	Yes
*F*‐statistic on instrument	159.1	103.6	56.33	490	338.5	155.4	443.7	101.0
Estimated first stage effect	0.64	0.60	0.63	0.52	0.66	0.64	0.73	0.61
No. of observations	22,466,784	14,488,379	6,170,349	8,317,345	9,209,007	27,746,216	5,850,526	31,104,700

*Note:* (1) HCBS: home‐ and community‐based services. (2) CIHHC: community‐initiated home health care. (3) TM: traditional Medicare. (4) MA: Medicare Advantage. (5) Stratified analysis by Medicare‐Medicaid integrated care plans enrollment is performed among Medicare Advantage enrollees. Medicare‐Medicaid integrated care plans include Dual Eligible Special Needs Plans (D‐SNPs) and Financial Alignment Initiative (FAI) demonstrations. (6) ESRD: end‐stage renal disease. (7) County‐pair‐year fixed effects: state‐border county‐pair specific year fixed effects. (8) Standard errors clustered at state level and state‐border county pair level separately are included in parentheses. (9) *p < 0.10, **p < 0.05, ***p < 0.01.

When examining heterogeneity by living arrangements and availability of non‐agency caregivers, we find that Medicaid HCBS use decreases Medicare CIHHC utilization by approximately 0.53 percentage points for older adults living alone in private homes with around‐the‐clock assistance (Appendix Table 9). This effect accounts for more than 50% of the reduction found in the main analysis. In contrast, while Medicaid HCBS use is estimated to reduce other categories of Medicare CIHHC utilization, these effects are substantially smaller in magnitude and are not statistically significant. These results are consistent with our hypothesis that Medicaid HCBS is more likely to reduce Medicare CIHHC utilization for older adults with greater LTSS needs.

### 3.4 Robustness Checks

When regressing Medicare CIHHC use on the instrument in a sample of non‐dual‐eligible older adults, we find a small and statistically insignificant coefficient, suggesting that the instrument is unlikely to affect Medicare CIHHC use through channels other than Medicaid HCBS use (Appendix Table 10).

Appendix Table 11 reports the results from the analysis using the all‐counties sample. The OLS results are similar to those from the main analysis in both direction and effect size. While the 2SLS results also suggest that Medicaid HCBS use decreases Medicare CIHHC utilization, the estimated effect is smaller than in the main analysis and is not statistically significant. It is not surprising that the effect is weaker in this analysis, as the instrument is likely correlated with other time‐varying county‐level confounders that could also affect Medicare CIHHC utilization. However, these results are qualitatively similar to those from the main analysis, suggesting that our findings may be applicable beyond state‐border counties. Finally, we present results from the analysis using a subsample that excludes states with limitations in data quality of Medicaid HCBS claims in Appendix Table 12. These results are consistent with our main findings in both direction and magnitude, suggesting that data quality limitations in these states do not materially affect our results.

## 4 Discussion

### 4.1 Conclusion

This study examines how Medicaid HCBS use influences the utilization of Medicare home health care initiated from the community without a prior institutional stay. Using an instrumental variable approach to address selection bias, we find that Medicaid HCBS use decreases Medicare CIHHC utilization by approximately 1.02 percentage points, representing a decrease of about 44% from the sample mean. In the first quarter of 2019, there were approximately 50 Medicare CIHHC episodes per 1000 dual‐eligible older adults [6], and the average Medicare payment per episode was $3039 [1]. A back‐of‐the‐envelope calculation suggests that this estimate may translate into reductions of approximately 22 Medicare CIHHC episodes and $66,858 in Medicare spending per 1000 dual‐eligible beneficiaries. This effect is heterogeneous across patient characteristics, as the reduction in Medicare CIHHC use is only found among enrollees of Medicare‐Medicaid ICPs and those that live in urban counties. In addition, we find that Medicaid HCBS use mainly reduces Medicare CIHHC used by individuals who live alone in private homes and receive around‐the‐clock assistance.

These findings suggest a substitution between Medicaid HCBS and Medicare CIHHC among dual‐eligible older adults at the margin of using Medicaid HCBS, namely those who will use Medicaid HCBS only if they live in states with Medicaid LTSS policies and infrastructure that favor home‐based care. Moreover, this substitution effect is strongest among older adults who live alone and receive around‐the‐clock assistance from private‐paid or informal caregivers, a group that is likely to have greater needs for publicly funded LTSS. While we cannot determine whether this substitution reflects a deliberate decision, we find that Medicaid HCBS and Medicare CIHHC operate as substitutes in a quantitative sense. These results align with our hypothesis that Medicare home health is being used as a substitute for long‐term care, and the availability of formal home‐based LTSS may help to alleviate this potentially inefficient use among dual‐eligible older adults. Additionally, we show that the reduction in Medicare CIHHC utilization is concentrated among enrollees of Medicare‐Medicaid ICPs, which have the financial incentive and structure to provide more coordinated care across the two programs, thus prompting plans to optimize the use of home‐based care for individuals with long‐term care needs. In contrast, dual‐eligible older adults who enrolled in TM or nonintegrated MA plans are responsible for navigating and coordinating benefits across Medicare and Medicaid on their own, which can be challenging for individuals without relevant institutional knowledge and expertise.

### 4.2 Limitations

Our study has several limitations. First, we are unable to incorporate health or clinical characteristics in the analysis because these data are only available for beneficiaries who used Medicare CIHHC or Medicaid HCBS. Since our sample includes year‐quarters for people both with and without service use, these characteristics are not available for all observations. Given our instrumental variable approach, omitting these characteristics only introduces bias if they vary systematically across levels of the instrument as well as the outcome, a violation of the assumptions of the model. We believe this to be unlikely, as all observed characteristics are well‐balanced across the instrument (Appendix Table 8). Nonetheless, we cannot rule out that some aspects of omitted health status, if they are uncorrelated with those observed characteristics, might cause bias in the estimation. Second, we measure Medicaid HCBS use at the individual‐quarter level, yet beneficiaries might have varying duration of use or may use multiple types of services in a quarter. In this study, we are unable to explore whether the relationship between Medicaid HCBS and Medicare CIHHC varies by intensity of use or by type of HCBS. Third, we cannot identify the availability of informal caregivers in our data. Informal caregivers could influence the use of both Medicaid HCBS and Medicare CIHHC, and future research should explore ways to incorporate this factor. Fourth, our study design may not fully capture longer term relationships between the two programs. For example, anticipated future need for LTSS could influence earlier Medicaid HCBS enrollment decisions. Fifth, our identification strategy yields a local average treatment effect, which applies only to dual‐eligible older adults living in state‐border counties who are at the margin of using Medicaid HCBS. Nevertheless, the characteristics of the state‐border county‐pair sample (Table 1) are similar to those of the all‐counties sample (Appendix Table 5), suggesting that our estimates may be informative for a broader population. Lastly, given the heterogeneity in state HCBS eligibility, coverage, payment and delivery policies, the nature and extent of effects, if any, may vary across states.

### 4.3 Policy Implications

Results from this study have important policy implications. First, our findings suggest that greater availability of Medicaid HCBS reduces Medicare CIHHC utilization among dual‐eligible beneficiaries at the margin of using HCBS. Our back‐of‐the‐envelope calculation implies that Medicaid HCBS induces savings for Medicare that are currently not considered in policy debates and that further state expansion of HCBS could lead to additional savings for Medicare. However, states' incentive for considering this synergy is limited if they do not capture Medicare savings. In addition, further expansion of Medicaid HCBS would still only benefit those who qualify for Medicaid, leaving most older adults uncovered. Importantly, among MA enrollees, we find that Medicaid HCBS use reduces Medicare CIHHC utilization mainly among older adults enrolled in ICPs. This is likely because such plans offer enrollees streamlined procedures and greater access to benefits from both programs, thereby improving the efficiency of care [26], aligned with plan incentives to reduce utilization and costs. Our findings thus support the expansion of integrated care models to strengthen care coordination for dual‐eligible beneficiaries.

Second, our findings on substitution between Medicaid HCBS and Medicare CIHHC among dual‐eligible older adults provide insight into the hypothesis that Medicare home health care may be used as a potential substitute for long‐term care among non‐dual‐eligible older adults. Under this hypothesis, one implication might be the need to strengthen and clarify rules around Medicare home health use, but this would not address the underlying problem of unmet need for LTSS. A 2019 report found that over 70% of older adults develop severe long‐term care needs, yet only 48% of them receive some paid care, with many of them relying on unpaid care from informal caregivers [27]. To address these unmet needs more directly, researchers and policymakers could jointly evaluate whether the current Medicare home health benefit could be adapted to better support certain LTSS needs. In the long run, to more completely address unmet LTSS needs and eliminate misaligned incentives across programs, Congress could consider establishing a new Medicare benefit dedicated solely to providing beneficiaries with access to long‐term care [28].

## Funding

This work was supported by the National Institute on Aging, 1RF1AG069857.

## Conflicts of Interest

The authors declare no conflicts of interest.

## Supporting information


**Appendix Figure 1** Flow diagram of study sample construction.
**Appendix Figure 2**. Trend in the utilization of Medicaid HCBS in all‐counties sample.
**Appendix Table 1**. Summary of the literature review on the relationship between Medicaid HCBS and Medicare CIHHC.
**Appendix Table 2**. The T‐MSIS Analytic Files (TAF) Analysis Reporting Checklist.
**Appendix Table 3**. Sample size and mean rates of Medicaid HCBS use by state.
**Appendix Table 4**. Data sources and data elements used to identify Medicaid HCBS.
**Appendix Table 5**. Characteristics of the study sample by Medicaid HCBS user status in all‐counties sample.
**Appendix Table 6**. Average Medicare CIHHC use by Medicaid HCBS user status.
**Appendix Table 7**. Summary statistics of the instrument, proportion of prevalent Medicaid HCBS users among dual‐eligible older adults in other counties within the same state in the previous quarter, for Medicaid HCBS use.
**Appendix Table 8**. Balance of observed county‐year‐level characteristics by the instrument, proportion of prevalent Medicaid HCBS users among dual‐eligible older adults in other counties within the same state in the previous quarter.
**Appendix Table 9**. Effects of Medicaid HCBS use on the utilization of Medicare CIHHC estimated using two‐stage least squares, classified by living arrangements and availability of around‐the‐clock assistance.
**Appendix Table 10**. Falsification test: effect of the instrumental variable on the utilization of Medicare CIHHC among non‐dually eligible beneficiaries, estimated using ordinary least squares.
**Appendix Table 11**. Effects of Medicaid HCBS use on the utilization of Medicare CIHHC, estimated using ordinary least squares and two‐stage least squares in all‐counties sample.
**Appendix Table 12**. Effects of Medicaid HCBS use on the utilization of Medicare CIHHC, estimated using two‐stage least squares in a sample excluding states with lower‐quality Medicaid HCBS claims.

## Data Availability

The data that support the findings of this study are available from Centers for Medicare & Medicaid Services. Restrictions apply to the availability of these data, which were used under license for this study. Data are available from the author(s) with the permission of Centers for Medicare & Medicaid Services.
