# Supplementary material for: Is Medicare Home Health Care Utilization Substituting for Long‐Term Care? Evidence From Dual Eligible Beneficiaries
Source: Health Serv Res. 2026 Apr 1;61(2):e70109. doi: 10.1111/1475-6773.70109 (PMC13042937; doi:10.1111/1475-6773.70109)
Supplement: Supplementary file 1 — Appendix Figure 1 Flow diagram of study sample construction. Appendix Figure 2. Trend in the utilization of Medicaid HCBS in all‐counties sample. Appendix Table 1. Summary of the literature review on the relationship between Medicaid HCBS and Medicare CIHHC. Appendix Table 2. The T‐MSIS Analytic Files (TAF) Analysis Reporting Checklist. Appendix Table 3. Sample size and mean rates of Medicaid HCBS use by state. Appendix Table 4. Data sources and data elements used to identify Medicaid HCBS. Appendix Table 5. Characteristics of the study sample by Medicaid HCBS user status in all‐counties sample. Appendix Table 6. Average Medicare CIHHC use by Medicaid HCBS user status. Appendix Table 7. Summary statistics of the instrument, proportion of prevalent Medicaid HCBS users among dual‐eligible older adults in other counties within the same state in the previous quarter, for Medicaid HCBS use. Appendix Table 8. Balance of observed county‐year‐level characteristics by the instrument, proportion of prevalent Medicaid HCBS users among dual‐eligible older adults in other counties within the same state in the previous quarter. Appendix Table 9. Effects of Medicaid HCBS use on the utilization of Medicare CIHHC estimated using two‐stage least squares, classified by living arrangements and availability of around‐the‐clock assistance. Appendix Table 10. Falsification test: effect of the instrumental variable on the utilization of Medicare CIHHC among non‐dually eligible beneficiaries, estimated using ordinary least squares. Appendix Table 11. Effects of Medicaid HCBS use on the utilization of Medicare CIHHC, estimated using ordinary least squares and two‐stage least squares in all‐counties sample. Appendix Table 12. Effects of Medicaid HCBS use on the utilization of Medicare CIHHC, estimated using two‐stage least squares in a sample excluding states with lower‐quality Medicaid HCBS claims. [file HESR-61-0-s001.docx]

Appendix Figure 1. Flow diagram of study sample construction


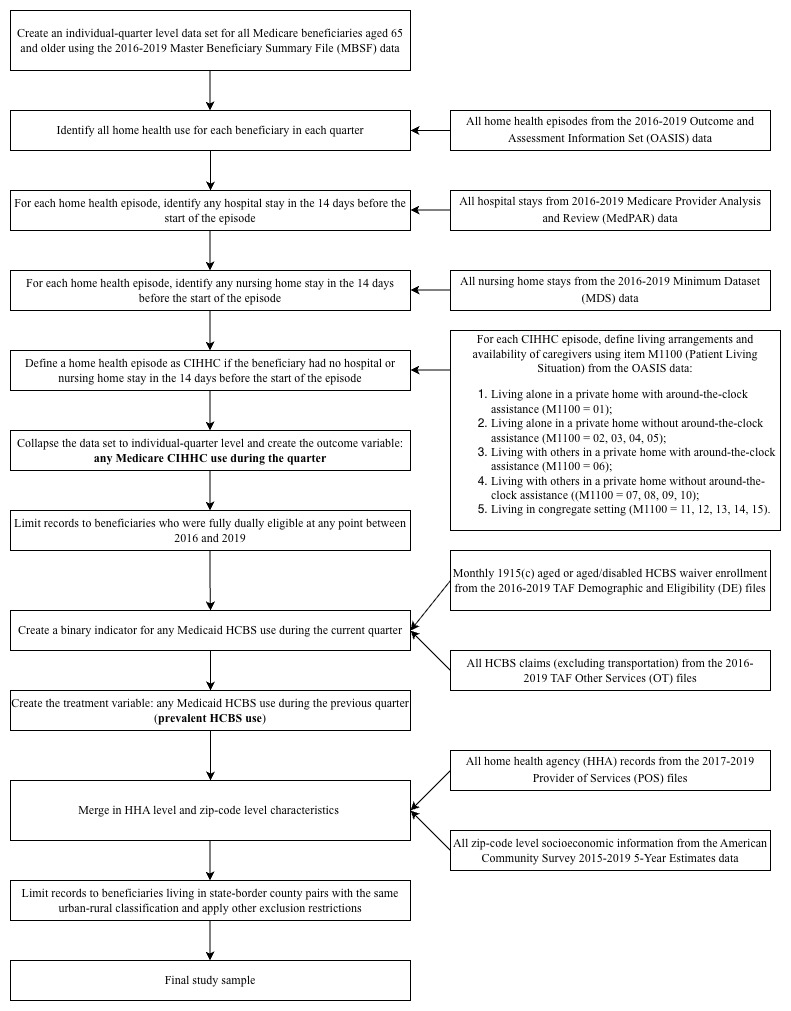


Note: (1) CIHHC: community-initiated home health care. (2) HCBS: home- and community-based services. (3) TAF: Transformed Medicaid Statistical Information System Analytic Files.

Appendix Figure 2. Trend in the utilization of Medicaid HCBS in all-counties sample


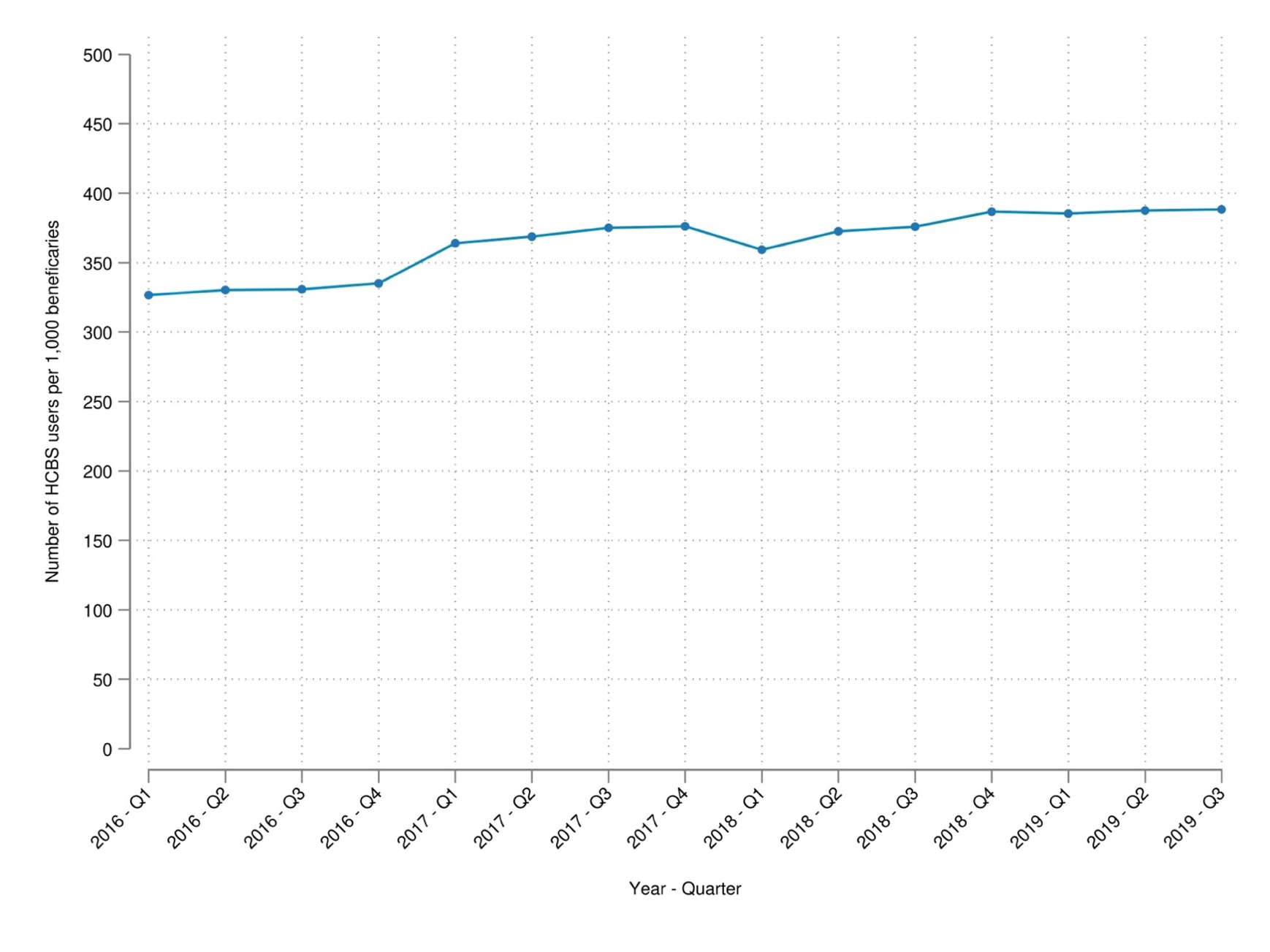


HCBS: home- and community-based services

Appendix Table 1. Summary of the literature review on the relationship between Medicaid HCBS and Medicare CIHHC

| Research question | How does the use of Medicaid HCBS affect Medicare CIHHC utilization among dual-eligible older adults? |
| --- | --- |
| Database | PubMed and Google Scholar |
| Keywords | Medicare community-initiated home health; Medicaid HCBS |
| Time period | 2001-2025 |
| Geographic boundaries | United States |
| Inclusion criteria | Studies that analyze Medicare CIHHC use or examine the relationship between Medicaid HCBS and Medicare home health care. |
| Exclusion criteria | 1. Studies which focus only on Medicaid HCBS without addressing Medicare home health care. 2. Studies using data prior to 2001. |
| Total number of studies found | 7 |
| Total number of studies reviewed | 7 |
| Studies reviewed | 1. Kim S, Qi M, Konetzka RT, Werner RM. Home Health Care Use Among Medicare Beneficiaries From 2010 to 2020. Med Care Res Rev. 2025;82(3):260-268. doi:10.1177/10775587251318407 2. Wang S, Werner RM, Coe NB, Chua R, Qi M, Konetzka RT. The role of Medicaid home- and community-based services in use of Medicare post-acute care. Health Services Research. 2024;59(5):e14325. doi:10.1111/1475-6773.14325 3. Fout B, Plotzke M, Jung OS. Heterogeneity in Medicare Home Health Patients by Admission Source. Home Health Care Management & Practice. 2019;31(1):9-15. doi:10.1177/1084822318793882 4. Burgdorf JG, Mroz TM, Wolff JL. Social Vulnerability and Medical Complexity Among Medicare Beneficiaries Receiving Home Health Without Prior Hospitalization. Sands LP, ed. Innovation in Aging. 2020;4(6):igaa049. doi:10.1093/geroni/igaa049 5. Burgdorf JG, Ornstein KA, Liu B, et al. Variation in Home Healthcare Use by Dementia Status Among a National Cohort of Older Adults. Lipsitz LA, ed. The Journals of Gerontology, Series A: Biological Sciences and Medical Sciences. 2024;79(3):glad270. doi:10.1093/gerona/glad270 6. Salant I, Shepard M, Maestas N, Layton TJ, Grabowski DC. Community-Entry Home Health Made Up Nearly Half Of Home Health Episodes And Spending In Traditional Medicare, 2017–21. Health Affairs. 2026;45(1):84-91. doi:[10.1377/hlthaff.2025.00318](https://doi.org/10.1377/hlthaff.2025.00318) 7. Wysocki A, Cheh V. Patterns of Care and Home Health Utilization for Community-Admitted Medicare Patients. Mathematica Inc.; 2019. Accessed August 10, 2025. http://aspe.hhs.gov/reports/patterns-care-home-health-utilization-community-admitted-medicare-patients-0 |
| Major findings | 1. The increase in Medicare CIHHC use between 2010 and 2020 was driven by growth among Medicare Advantage enrollees who were not dual eligible (Kim et al., 2025). 2. Dual-eligible beneficiaries using Medicaid HCBS had higher use of home health care relative to nursing home care, conditional on receiving PAC (Wang et al., 2024). 3. Medicare CIHHC users are older, are more likely to be dual-eligible, have multiple home health episodes, have Alzheimer's disease, and have greater needs for informal caregiving compared to those referred to home health from a hospital or a skilled nursing facility (Burgdorf et al., 2020; Burgdort et al., 2024; Fout et al., 2019; Salant et al., 2026). 4. Based on descriptive statistics, there is no evidence suggesting that states increasingly using the Medicare home health benefit as a substitute for Medicaid LTSS (Wysocki and Cheh, 2019). 5. Overall, to the best of our knowledge, no prior study has examined the causal relationship between Medicaid HCBS and Medicare CIHHC among dual-eligible older adults. |

Note: (1) HCBS: home- and community-based services. (2) CIHHC: community-initiated home health care.

Appendix Table 2. The T-MSIS Analytic Files (TAF) Analysis Reporting Checklist

| **Category** | **Description** |
| --- | --- |
| **Data** |  |
| Files, Years, Release Version, and Data Extract | We use 100% TAF Demographic and Eligibility files and TAF Other Services files for 2016-2018 (Release 2) and 2019 (Release 1). |
| **Analytic Sample** |  |
| Eligibility Criteria | We limit our analysis to individuals aged 65 and older. |
| Enrollment Span | We construct our study sample at the individual-quarter level. For each quarter, we require individuals to be continuously enrolled in Medicaid throughout the quarter. |
| Scope of benefits | Our sample is restricted to individuals with full-scope benefits. |
| Encounter Data | N/A |
| Dual Eligibility | We limit our analysis to individuals with full dual eligibility throughout the quarter. |
| **State and territory exclusions** |  |
| Criteria | Our sample is restricted to contiguous state-border counties, as described in Section 2.2. Alaska, Hawaii, and the District of Columbia are excluded based on this criterion. |
| State variation table | We summarize the sample size and mean rates of Medicaid HCBS use by state in the study sample in Appendix Table 3. |
| **Special considerations** |  |
| Spending | N/A |
| Using TAF with predecessor MAX data | N/A |

Appendix Table 3. Sample size and mean rates of Medicaid HCBS use by state

| **State** | **N** | **Mean** |
| --- | --- | --- |
| Alabama | 197,678 | 0.25 |
| Arizona | 425,843 | 0.12 |
| Arkansas | 363,158 | 0.32 |
| California | 1,417,754 | 0.25 |
| Colorado | 172,844 | 0.43 |
| Connecticut | 787,447 | 0.35 |
| Delaware | 533,518 | 0.20 |
| Florida | 79,488 | 0.14 |
| Georgia | 326,482 | 0.17 |
| Idaho | 102,125 | 0.29 |
| Illinois | 3,607,389 | 0.31 |
| Indiana | 619,000 | 0.19 |
| Iowa | 222,937 | 0.25 |
| Kansas | 222,947 | 0.33 |
| Kentucky | 532,776 | 0.17 |
| Louisiana | 324,780 | 0.19 |
| Maine | 114,798 | 0.06 |
| Maryland | 1,291,270 | 0.15 |
| Massachusetts | 3,017,856 | 0.36 |
| Michigan | 241,071 | 0.19 |
| Minnesota | 262,820 | 0.51 |
| Mississippi | 380,019 | 0.32 |
| Missouri | 1,230,828 | 0.32 |
| Montana | 62,484 | 0.24 |
| Nebraska | 190,075 | 0.64 |
| Nevada | 488,176 | 0.28 |
| New Hampshire | 295,779 | 0.21 |
| New Jersey | 2,303,076 | 0.24 |
| New Mexico | 331,161 | 0.27 |
| New York | 5,756,588 | 0.32 |
| North Carolina | 869,509 | 0.80 |
| North Dakota | 57,179 | 0.24 |
| Ohio | 1,151,472 | 0.28 |
| Oklahoma | 226,025 | 0.28 |
| Oregon | 401,924 | 0.38 |
| Pennsylvania | 3,709,657 | 0.24 |
| Rhode Island | 796,276 | 0.13 |
| South Carolina | 525,455 | 0.24 |
| South Dakota | 72,700 | 0.23 |
| Tennessee | 966,376 | 0.15 |
| Texas | 393,347 | 0.58 |
| Utah | 60,560 | 0.15 |
| Vermont | 92,747 | 0.27 |
| Virginia | 836,940 | 0.20 |
| Washington | 263,379 | 0.47 |
| West Virginia | 296,697 | 0.27 |
| Wisconsin | 259,450 | 0.34 |
| Wyoming | 73,366 | 0.24 |

Note: (1) The sample size and mean rates are summarized at the beneficiary-quarter level in the state-border county-pair sample. (2) HCBS: home- and community-based services.

Appendix Table 4. Data sources and data elements used to identify Medicaid HCBS

| Data source | Data element | | Values |
| --- | --- | --- | --- |
| TAF Demographic and Eligibility file | Waiver type Code | | 06, 07, 08, 09, 10, 11, 12, 13, 14, 15, 16, 17, 18, 19, 20, 33 |
| TAF Other Services file | Type of service code | Type of service code | 016, 017, 018, 019, 020, 021, 036, 043, 051, 053, 054, 062, 063, 064, 065, 066, 067, 068, 069, 070, 071, 072, 073, 074, 075, 076, 077, 078, 079, 080, 081, 082, 083, 115 |
|  |  | Type of service code and place of service code | 022, 087 and place of service code of 12 |
|  | Benefit type code | Benefit type code | 015, 016, 017, 022, 068, 076, 043, 036, 042, 077, 085, 078, 079, 080, 081, 082, 083, 084 |
|  |  | Benefit type code and place of service | 023, 069, 041 and place of service code of 12 |

Note: (1) HCBS: home- and community-based services. (2) TAF: Transformed Medicaid Statistical Information System Analytic Files.

Appendix Table 5. Characteristics of the study sample by Medicaid HCBS user status in all-counties sample

|  | All | Non-HCBS users | HCBS users |
| --- | --- | --- | --- |
|  | N = 66,212,039 | N = 46,398,613 | N = 19,813,426 |
| Age, mean (SD) | 76.88 (8.54) | 76.36 (8.50) | 78.11 (8.49) |
| Female, % | 65.83 | 64.08 | 69.91 |
| Race and ethnicity, % |  |  |  |
| - White | 58.23 | 59.32 | 55.68 |
| - Black or African American | 17.00 | 16.16 | 18.98 |
| - Hispanic | 10.49 | 10.97 | 9.36 |
| - Other | 14.28 | 13.55 | 15.99 |
| Aged into Medicare, % | 76.68 | 79.29 | 70.58 |
| Enrolled in Medicare Advantage, % | 40.95 | 42.86 | 36.47 |
| Eligible days for Medicare CIHHC use, mean (SD) | 88.83 (8.47) | 89.07 (8.07) | 88.26 (9.31) |
| Median household income of the zip code, mean (SD) | 60,699.56 (25,377.88) | 61,229.38 (25,463.55) | 59,458.84 (25,132.45) |
| Percent of zip code population of age 65 and over, mean (SD) | 15.96 (5.96) | 16.14 (6.13) | 15.55 (5.53) |
| Percent of zip code population of age 65 and over that are living in poverty, mean (SD) | 12.83 (8.24) | 12.48 (8.02) | 13.66 (8.66) |
| Percent of zip code population of age 65 and over with private health insurance, mean (SD) | 50.82 (16.08) | 51.31 (16.11) | 49.65 (15.97) |
| Number of HHAs in the county per 1,000 Medicare beneficiaries, mean (SD) | 0.49 (0.51) | 0.46 (0.46) | 0.55 (0.60) |
| Number of RNs in the county per 1,000 Medicare beneficiaries, mean (SD) | 4.13 (6.84) | 4.12 (7.22) | 4.16 (5.85) |
| Number of home health aides in the county per 1,000 Medicare beneficiaries, mean (SD) | 2.77 (7.00) | 2.69 (6.89) | 2.96 (7.26) |
| Rural county, % | 15.81 | 15.49 | 16.55 |

Note: (1) The characteristics are summarized at the beneficiary-quarter level. (2) HCBS: home- and community-based services. (3) CIHHC: community-initiated home health care. (4) HHA: home health agency. (5) RN: registered nurse.

Appendix Table 6. Average Medicare CIHHC use by Medicaid HCBS user status

|  | Non-HCBS users | HCBS users |
| --- | --- | --- |
|  | N = 26,234,565 | N = 10,720,661 |
| Medicare CIHHC use, % | 1.54 | 4.15 |

Note: (1) The outcome is summarized at the beneficiary-quarter level. (2) CIHHC: community-initiated home health care. (3) HCBS: home- and community-based services.

Appendix Table 7. Summary statistics of the instrument, proportion of prevalent Medicaid HCBS users among dual-eligible older adults in other counties within the same state in the previous quarter, for Medicaid HCBS use

|  | Medicaid HCBS use, mean (SD) |
| --- | --- |
| Observations within the lowest quartile of the instrument | 0.19 (0.39) |
| Observations within the 2^nd^ quartile of the instrument | 0.24 (0.42) |
| Observations within the 3^rd^ quartile of the instrument | 0.30 (0.46) |
| Observations within the highest quartile of the instrument | 0.44 (0.50) |
| No. of observations | 36,955,226 |

Note: (1) HCBS: home- and community-based services.

Appendix Table 8. Balance of observed county-year-level characteristics by the instrument, proportion of prevalent Medicaid HCBS users among dual-eligible older adults in other counties within the same state in the previous quarter

|  | All counties | Counties with lower values of the instrument | Counties with higher values of the instrument |
| --- | --- | --- | --- |
|  | N = 7,072 | N = 3,536 | N = 3,536 |
| Age, mean (SD) | 77.75 (1.93) | 77.70 (2.00) | 77.81 (1.85) |
| Female, % | 68.18 | 68.33 | 68.04 |
| Race and ethnicity, % |  |  |  |
| - White | 80.71 | 80.51 | 80.91 |
| - Black or African American | 11.50 | 11.37 | 11.63 |
| - Hispanic | 2.74 | 2.59 | 2.89 |
| - Other | 5.05 | 5.53 | 4.58 |
| Aged into Medicare, % | 71.59 | 71.52 | 71.66 |
| Enrolled in Medicare Advantage, % | 21.44 | 20.89 | 21.99 |
| Eligible days for Medicare CIHHC use, mean (SD) | 88.52 (0.91) | 88.50 (0.91) | 88.54 (0.90) |
| Median household income of the zip code, mean (SD) | 52,126.91 (14804.07) | 52,276.15 (14716.88) | 51,977.67 (14891.34) |
| Percent of zip code population of age 65 and over, mean (SD) | 19.33 (4.54) | 19.30 (4.55) | 19.37 (4.53) |
| Percent of zip code population of age 65 and over that are living in poverty, mean (SD) | 10.57 (4.76) | 10.60 (5.06) | 10.54 (4.44) |
| Percent of zip code population of age 65 and over with private health insurance, mean (SD) | 57.79 (9.16) | 57.82 (9.20) | 57.77 (9.12) |
| Number of HHAs in the county per 1,000 Medicare beneficiaries, mean (SD) | 5.57 (31.04) | 6.20 (43.71) | 4.94 (3.94) |
| Number of RNs in the county per 1,000 Medicare beneficiaries, mean (SD) | 2.88 (12.60) | 3.23 (17.30) | 2.53 (4.23) |
| Number of home health aides in the county per 1,000 Medicare beneficiaries, mean (SD) | 1.93 (5.08) | 2.04 (6.02) | 1.83 (3.93) |
| Rural county, % | 69.12 | 69.12 | 69.12 |

Note: (1) HCBS: home- and community-based services. (2) CIHHC: community-initiated home health care. (3) HHA: home health agency. (4) RN: registered nurse.

Appendix Table 9. Effects of Medicaid HCBS use on the utilization of Medicare CIHHC estimated using two-stage least squares, classified by living arrangements and availability of around-the-clock assistance

|  | Living alone with around-the-clock assistance | Living alone without around-the-clock assistance | Living with others with around-the-clock assistance | Living with others without around-the-clock assistance | Living in congregate setting |
| --- | --- | --- | --- | --- | --- |
| Estimated effect | -0.0053^***^  (0.002) | -0.0007  (0.0018) | -0.0027  (0.0018) | -0.0006  (0.0007) | -0.001  (0.0009) |
| Sample mean | 0.0024 | 0.0054 | 0.0095 | 0.0025 | 0.0025 |
| County fixed effects | Yes | Yes | Yes | Yes | Yes |
| County-pair-year fixed effects | Yes | Yes | Yes | Yes | Yes |
| Quarter fixed effects | Yes | Yes | Yes | Yes | Yes |
| *F*-statistic on instrument | 173.6 | 173.6 | 173.6 | 173.6 | 173.6 |
| Estimated first stage effect | 0.65 | 0.65 | 0.65 | 0.65 | 0.65 |
| No. of observations | 36,955,226 | 36,955,226 | 36,955,226 | 36,955,226 | 36,955,226 |

Note: (1) HCBS: home- and community-based services. (2) CIHHC: community-initiated home health care. (3) Information on patients’ living arrangements and availability of assistance is determined based on item M1100 (Patient Living Situation) in the Outcome and Assessment Information Set (OASIS). (4) County-pair-year fixed effects: state-border county-pair specific year fixed effects. (5) Standard errors clustered at state level and state-border county pair level separately are included in parentheses. (6) ^*^ p<0.10, ^**^ p<0.05, ^***^ p<0.01.

Appendix Table 10. Falsification test: effect of the instrumental variable on the utilization of Medicare CIHHC among non-dually eligible beneficiaries, estimated using ordinary least squares

|  | OLS |
| --- | --- |
| Estimated effect | -0.00096  (0.0006) |
| Sample mean | 0.0089 |
| County fixed effects | Yes |
| County-pair-year fixed effects | Yes |
| Quarter fixed effects | Yes |
| No. of observations | 286,009,118 |

Note: (1) Instrumental variable: proportion of prevalent Medicaid HCBS users among dual-eligible beneficiaries in other counties within the same state in the previous quarter. (2) CIHHC: community-initiated home health care. (3) OLS: ordinary least squares. (4) County-pair-year fixed effects: state-border county-pair specific year fixed effects. (5) Standard errors clustered at state level and state-border county-pair level separately are included in parentheses. (6) ^*^ p<0.10, ^**^ p<0.05, ^***^ p<0.01.

Appendix Table 11. Effects of Medicaid HCBS use on the utilization of Medicare CIHHC, estimated using ordinary least squares and two-stage least squares in all-counties sample

|  | OLS | Reduced form | 2SLS |
| --- | --- | --- | --- |
| Estimated effect | 0.026^***^  (0.0037) | -0.0047  (0.0052) | -0.0063  (0.0068) |
| Sample mean | 0.025 | 0.025 | 0.025 |
| County fixed effects | Yes | Yes | Yes |
| Year fixed effects | Yes | Yes | Yes |
| Quarter fixed effects | Yes | Yes | Yes |
| *F*-statistic on instrument | − | − | 572.7 |
| Estimated first stage effect | − | − | 0.75 |
| No. of observations | 66,212,039 | 66,212,039 | 66,212,039 |

Note: (1) HCBS: home- and community-based services. (2) CIHHC: community-initiated home health care. (3) OLS: ordinary least squares. (4) Reduced form: regression of Medicare CIHHC utilization directly on the instrumental variable. (5) 2SLS: two-stage least squares. (6) Standard errors clustered at state level are included in parentheses. (7) ^*^ p<0.10, ^**^ p<0.05, ^***^ p<0.01.

Appendix Table 12. Effects of Medicaid HCBS use on the utilization of Medicare CIHHC, estimated using two-stage least squares in a sample excluding states with lower-quality Medicaid HCBS claims

|  | OLS | Reduced form | 2SLS |
| --- | --- | --- | --- |
| Estimated effect | 0.0253^***^  (0.0052) | -0.0082^***^  (0.0026) | -0.0131^***^  (0.0042) |
| Sample mean | 0.023 | 0.023 | 0.023 |
| County fixed effects | Yes | Yes | Yes |
| County-pair-year fixed effects | Yes | Yes | Yes |
| Quarter fixed effects | Yes | Yes | Yes |
| *F*-statistic on instrument | − | − | 104.2 |
| Estimated first stage effect | − | − | 0.63 |
| No. of observations | 34,348,973 | 34,348,973 | 34,348,973 |

Note: (1) HCBS: home- and community-based services. (2) CIHHC: community-initiated home health care. (3) OLS: ordinary least squares. (4) Reduced form: regression of Medicare CIHHC utilization directly on the instrumental variable. (5) 2SLS: two-stage least squares. (6) States with lower-quality Medicaid HCBS claims: Alabama, Hawaii, Iowa, Nebraska, Nevada, South Carolina, Wisconsin, North Dakota, Oregon, and Washington. (7) Standard errors clustered at state level are included in parentheses. (8) ^*^ p<0.10, ^**^ p<0.05, ^***^ p<0.01.
